# Mueller Matrix Analysis of Collagen and Gelatin Containing Samples Towards More Objective Skin Tissue Diagnostics

**DOI:** 10.3390/polym12061400

**Published:** 2020-06-22

**Authors:** Dierk Fricke, Alexander Becker, Annice Heratizadeh, Sara Knigge, Lennart Jütte, Merve Wollweber, Thomas Werfel, Bernhard Wilhelm Roth, Birgit Glasmacher

**Affiliations:** 1 Hannover Centre for Optical Technologies (HOT), Leibniz University Hannover, 30167 Hannover, Germany; lennart.juette@hot.uni-hannover.de (L.J.); m.wollweber@lzh.de (M.W.); bernhard.roth@hot.uni-hannover.de (B.W.R.); 2 Institute for Multiphase Processes (IMP), Leibniz University Hannover, 30167 Hannover, Germany; becker@imp.uni-hannover.de (A.B.); knigge@imp.de (S.K.); glasmacher@imp.uni-hannover.de (B.G.); 3 Implant Research and Development (NIFE), Lower Saxony Centre for Biomedical Engineering, 30625 Hannover, Germany; 4 Hannover Medical School, Department of Dermatology and Allergy, 30625 Hannover, Germany; heratizadeh.annice@mh-hannover.de (A.H.); Werfel.Thomas@mh-hannover.de (T.W.); 5 Laser Zentrum Hannover e.V., 30419 Hannover, Germany; 6 Cluster of Excellence PhoenixD, Leibniz University Hannover, 30167 Hannover, Germany

**Keywords:** Mueller matrix, tissue engineering, electrospinning, fibre alignment, collagen, gelatin, Raman spectroscopy

## Abstract

Electrospun polycaprolactone:gelatin (PCL:GT) fibre scaffolds are widely employed in the field of tissue implants. Here, the orientation of fibres plays an important role in regard to implantation due to the impact on the mechanical properties. Likewise, the orientation of collagen fibres in skin tissue is relevant for dermatology. State-of-the-art fibre orientation measurement methods like electron microscopy are time consuming and destructive. In this work, we demonstrate polarimetry as a non-invasive approach and evaluate its potential by measuring the Mueller matrix (MM) of gelatin and collagen containing samples as simple skin tissue phantoms. We demonstrate that it is possible to determine the orientation of PCL:GT fibre scaffolds within one MM measurement. Furthermore, we determine the structural orientation in collagen film samples. Currently, the diagnosis of skin diseases is often performed by image analysis or histopathology respectively, which are either subjective or invasive. The method presented, here, provides an interesting alternative approach for such investigations. Our findings indicate that the orientation of collagen fibres within skin lesions might be detectable by MM measurements in the future, which is of interest for skin diagnostics, and will be further investigated during the next step.

## 1. Introduction

Non-invasive and fast measurement techniques are of interest for investigations in the life sciences, i.e., for medical diagnostics and tissue implant characterisation. Optical technologies have already indicated their application potential in these fields. In general, the assessment of tissue properties or disorders requires the reliable detection of subtle changes in tissue structure to be able to initiate measures for treatment in case of diseases. For example, inflammatory skin lesions occur in a broad variety of skin disorders with different pathogenesis. Typical hallmarks of clinically inflamed skin are redness, edema or induration (i.e., hardening and thickening) and scaling. In clinically uncertain cases, taking a skin biopsy for histopathological analysis is usually indicated. Conventional hand-held dermatoscopes serve as essential non-invasive diagnostic tools for evaluating pigmented lesions, but are currently not suitable for further examination of inflamed skin. Indeed, flattening of the skin lesion while using the conventional dermatoscope leads to changes or even reduction of the blood circulation and finally affects the disease-related color of the corresponding skin site. To avoid direct skin contact during examination we developed a prototype for a non-contact, remote digital dermatoscope in previous work [1,2]. In an initial study we attempted to visualise skin inflammation of common inflammatory skin diseases such as psoriasis and lichen planus by non-contact remote digital dermoscopy [3]. We revealed promising first results regarding a visualisation of inflammatory changes within epidermal, i.e., upper, layers of the skin by non-contact remote dermoscopy. It was possible to visualise disease-specific changes within the epidermis like an expansion of the granular layer in lichen planus. Simultaneously, the system generated true-color images of the skin lesion [3].

However, it remains a challenge to explore changes located deeper than the epidermis as relevant for inflammatory diseases. In patients with sclerodermia circumscripta and granuloma anulare, using the prototype it was challenging to generate optical signals reflecting a distinct disease-related pattern [3]. In patients with sclerodermia, affected skin lesions are clinically characterized by fibrosis and sclerosis, resulting in a hardening of the skin and hair loss because of a destruction of the hair follicles. Histopathology in sclerodermia circumscripta is characterized by a deposition of collagen in the dermis [4] and an edematous formation of collagen. Thus, for sufficient non-invasive examination of inflammatory skin disorders further development of non-contact remote digital dermatoscopy is required as they are located deeper in the skin with involvement of collagen formation, such as in patients with sclerodermia.

To generate additional diagnostically relevant information, different techniques are in principle possible for non-contact measurement. Common dermatoscopes are designed for 2D imaging based on digital imaging of the skin by using a charge-coupled device (CCD) or complementary metal–oxide–semiconductor (CMOS) chip. This technique provides spatially resolved spectral information. State-of-the-art camera devices have three different color channels. To generate further information, in theory the use of hyperspectral cameras with more color channels would be possible [5]. Additionally, optical coherence tomography (OCT) is a common technique for skin investigation with an imaging depth of 2–3 mm in most biological tissues and axial resolution between 1 and 15 µm [6]. However, the OCT signal is based only on the backscattering intensity from different depths and provides no spectral information [6]. Ultrasound also enables depth information based on the intensity of measured waves. In this case acoustic waves are used, and the depth is defined by the time the wave propagates in the target. The imaging depth is inversely proportional to the lateral resolution and the required frequency. In muscle tissue, for example, the lateral resolution can be as low as 40 µm [7]. Another approach to gain more information of possible diagnostic relevance is to use polarized light. Typically, a cross polarization setup is realized, where the sample is illuminated with linearly polarised light. Only light with polarisation perpendicular to the illumination is detected. Cross polarisation is used to suppress surface-reflected signals and, therefore, reveal information on structures below the surface. Even more information could be obtained by measuring the Mueller matrix (MM) of the target [8]. Fricke et al. devised a non-destructive, cost-efficient and contactless quantification method for the degree of fibre alignment in electrospun polycaprolactone (PCL) fibre scaffolds, based on Mueller matrix (MM) polarimetry [9].

Motivated by the described limitations of current approaches and to meet demands from dermatology, in this work, we investigate the potential of MM-measurement to obtain information about orientations within collagen and gelatin containing structures. These were chosen as simple tissue phantoms for our initial proof-of-principle investigations, the latter being realised by an electrospinning process, which is a versatile and scalable manufacturing technique ensuring high throughput and cost-efficiency. The method is widely used in tissue engineering (TE), to fabricate porous scaffolds based on fibres with diameters ranging from several hundred nanometers to a few microns. The results achieved indicate that the MM measurement provides valuable insights on tissue parameters, which are independent from fabrication- and application-based factors like scaffold thickness, fibre diameter or polymer type. Furthermore, in order to investigate the influence of the electrospinning process on the proteins in the blended solution and to confirm the collagen content in the transparent films, Raman spectroscopy was used. In the future, based on the results obtained here, we will investigate the suitability of our approach for skin diagnostics. In particular, we aim at differential diagnostics of inflammatory skin diseases in vivo.

## 2. Materials and Methods 

### 2.1. Electrospun PCL: Gelatin Fibre Scaffolds

**Figure 1:**
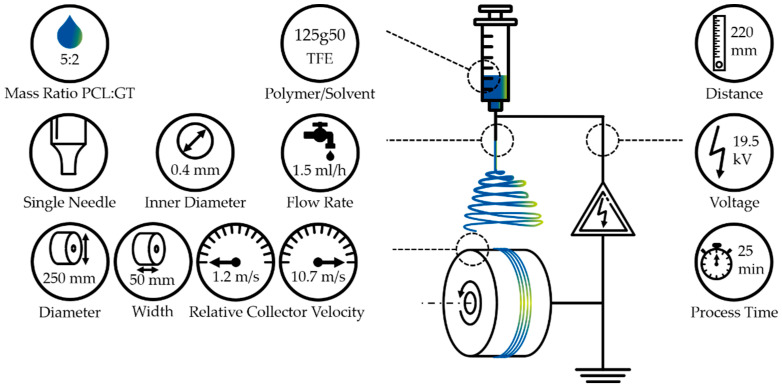
Overview of the experimental setup and adjusted process parameters. The electrospinning device used is composed of a grounded and rotating drum collector, high voltage supply, cannula, syringe and syringe pump. The adjusted parameters—the needle tip to collector distance, applied voltage, total process time, relative collector velocity, diameter and width of the drum collector, flow rate, inner cannula diameter, setup type, polymer and solvent as well as the mass ration of the used blend—are displayed.

In general, an electrospinning setup consists of an emitter, a high voltage supply and a grounded collector (see Figure 1). An initial droplet of polymeric solution is vertically emitted into an electrical field. As soon as the forces, induced by the electrical field and gravity, overcome the surface tension of the fluid, a fibre jet is emitted. On its way to the grounded collector, the fibre is constantly accelerated and the solvent evaporates. Upon deposition on the collector, the fibre diameter is constantly decreasing. The manufactured fibre scaffolds are strongly influenced by the ambient, process and solution parameters.

In tissue engineering an important task is to combine manufactured scaffolds, mimicking the extra cellular matrix (ECM), with cells and signaling factors. In the case of artificial skin substitutes, a variety of polymers have successfully been used as scaffold material [10]. The recreation of the ECM calls for specifically oriented fibres. The fibre orientation within the manufactured scaffold is mostly influenced by the relative velocity of the collector; the higher the relative collector velocity, the higher the degree of fibre alignment [9]. While this is not expected to be a linear relation, we anticipate an increase of the degree of alignment until a certain threshold and a stagnating or decreasing behavior after passing of this mark.

#### 2.1.1. Processing System

We used an electrospinning device according to Fricke et al. that consists of a drum collector, an electric motor, a blunt cannula, polyethylene tubing, a syringe and a syringe pump (see Supplementary Materials Figure S1) [9,11,12,13].

#### 2.1.2. Parameter Settings and Experimental Procedure

The fibre scaffolds were fabricated from a blended polymeric solution. The blends consisted of polycaprolactone (PCL, 80 kDa, Sigma-Aldrich Chemistry Corporate, St. Louis, MI, USA) and gelatin from bovine skin (Type B, ~225 g Bloom, Sigma-Aldrich Chemistry Corporate, St. Louis, MI, USA) in 2,2,2-trifluoroethanol (TFE, 99.8%, abcr GmbH, Karlsruhe, Germany) [14]. In order to enhance the solubility of gelatin in TFE, 2 v% of acetic acid were added. The mass ratio of PCL to gelatin was 5:2, resulting in 125 mg/mL PCL/TFE and 50 mg/mL gelatin/TFE. The polymeric solution preparation was conducted as previously published by Suresh et al. [14]. Three different fibre scaffolds were manufactured for each of the 13 relative collector velocities of 1.2 to 10.7 m/s at a total process duration of 25 min each (see Figure 1) [9,14]. An additional reference sample for the spectrographic analysis was fabricated from a 175 mg/mL PCL/TFE solution at a relative collector velocity of 2 m/s.

### 2.2. Collagen Film

The sample used was Viscofan Coffi Folie (Viscofan, Cáseda, Spain). It basically consists (in mass percent) of collagen (59–63.7%), water (15%), glycerin and Sorbitol (14–16%) and oil (4%) [15]. The product is supplied on a 38 cm wide roll of 50 m length. The film has a thickness of 0.04 mm.

### 2.3. Raman Spectroscopy

In order to assess the molecular structure and content of specific proteins as well as possible changes (manufacturing and handling), Raman spectroscopy (Alpha 300 RA, Witec GmbH, Ulm, Germany) was performed. The Raman spectrograph contained a laser with 532 nm wavelength and laser power adjusted to 30 mW and a CCD detector for spectra detection. It had an NA of 0.9 and spectral resolution down to 0.1 relative wavenumbers (at 633 nm). The integration time was set to 0.7 s as well as the accumulation to 50. The samples employed for the measurements were prepared from a collagen film, a pure PCL fibre scaffold and PCL:GT scaffolds fabricated with 1.2 m/s and 10.7 m/s relative collector velocity. Afterwards, the resulting spectra were normalized to the CH band intensity at 2923 cm^−1^. and compared.

### 2.4. SEM-Based Analysis

According to Fricke et al., SEM (S-3400N, Hitachi High-Tech Analytical Science Ltd., Tubney Woods, Abington, UK) images of sputter coated samples were taken.

#### 2.4.1. Fibre Scaffolds

The degree of fibre alignment was determined, based on nine images, three for each of the three samples per relative collector velocity. Subsequently, fibre orientation was measured by software analysis (AxioVision^®^, Carl Zeiss AG, Jena, Germany) [9].

#### 2.4.2. Collagen Film

Six pictures were taken, three of the upper and three of the lower side of the film. These images were investigated for potential fibre alignment and overall structure.

### 2.5. Mueller Matrix Measurement System

The Mueller matrix (MM) contains information about the polarisation changing properties of a sample. It can be calculated using intensity measurements where the sample investigated is illuminated with light of different polarisation states. The intensity for different polarisation states of the light after interaction with the sample is measured. The MM measurement system generates these states (with a polarisation state generator (PSG)) and filters the states of interest before sensing (with a polarisation state analyser (PSA)). As the sensor is a camera, 2D-measurements of the MM over a sample surface can be obtained. The MM measurement system used in this work was a self-built lab prototype described in detail in [9]. To calculate the full MM, 36 measurements of different combinations of illuminating and measured light had to be performed. The device generates and measures horizontal (H), vertical (V) +45° (P), −45° (M), right circular (R) and left circular (L) polarisation states as required for MM calculation. The PSG and PSA consist of a fixed polarizer and two liquid crystal retarders (LCR). By changing the voltage applied to the LCR, all required polarisation states can be generated in a comparably short time of 15 s. Potentially, the measurement time can be decreased to below 1 s in the future, since the system is switched entirely electronically without moving any parts. The system operates in reflection and transmission mode. The illumination is monochromatic and can be switched between 445 nm, 532 nm and 633 nm. To interpret the results, for example, polar decomposition of the MM can be performed [16]. The matrix is divided in three matrices of known physical properties. 

### 2.6. Statistical Analysis

SEM-based results were expressed as boxplots with outliers or means ± standard deviation (SD), if not stated otherwise [9]. In all boxplots, the interquartile range (IQR), represented by a closed box, was defined as 50% of the measured data. The line within the IQR was defined as the median value, while the displayed whiskers represented 1.5 times the IQR. Distance between whiskers was defined as the dispersion. The mean value was displayed as a rhombus. All values located outside of 1.5 × IQR were displayed as individual crosses. These values were “outliers” with regards to the IQR, but were not necessarily extreme values.

QQ-plots were generated to investigate the application of parametric or non-parametric tests. In order to determine differences between all groups, one-way repeated measures analysis of variance (ANOVA) was conducted. Subsequently, Tukey and Dunnett (reference: 1.2 m/s) post-hoc tests were performed to investigate differences between specific groups. Differences were considered significant at *p* < 0.05 (*), *p* < 0.01 (**) and *p* < 0.001 (***). All data were analysed using statistical analysis software (Origin 2018b, OriginLab Corporation, Northampton, Massachusetts, USA). 

## 3. Results

### 3.1. Raman Spectroscopy

**Figure 2:**
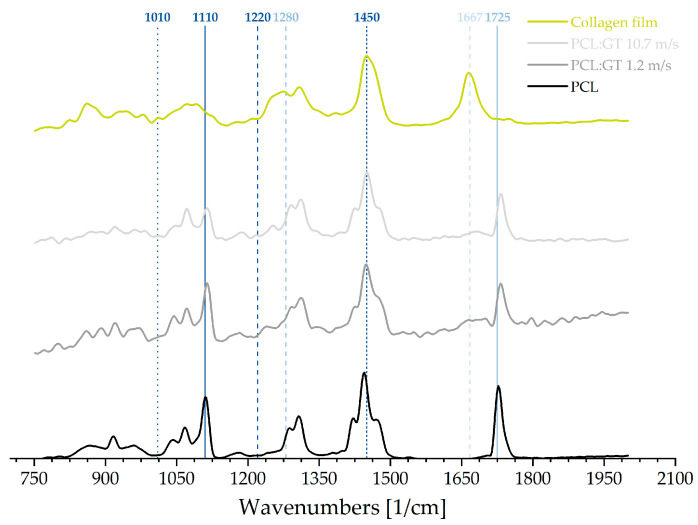
Raman spectra of pure PCL, PCL:GT 1.2 m/s, PCL:GT 10.7 m/s and collagen film. PCL is displayed in black, PCL:GT 1.2 m/s in dark grey, PCL:GT 10.7 m/s in light grey and collagen film in green. The vertical lines represent characteristic wavenumbers: phenylalanine (1010 cm^–1^; dotted dark blue), PCL (1110 cm^–1^; solid dark blue and 1725 cm^–1^; solid blue), amide (II: 1220 cm^–1^; dashed dark blue, III: 1280 cm^–1^; dashed blue and I: 1667 cm^–1^; dashed light blue) and lysine (1450 cm^–1^; small dashed dark blue), with the solid lines marking the PCL-typical bands and the dashed and dotted lines marking the protein typical bands.

The resulting Raman spectra of pure PCL as well as for the samples of PCL:GT (1.2 m/s and 10.7 m/s) and the collagen film are presented in Figure 2. Typical protein bands, like the prominent peak at 1667 cm^−1^ which could be associated with amide I vibration, as well as the broad band between 1220 cm^−1^ and 1280 cm^−1^, characterizing amide III, were clearly visible in the spectrum of collagen [17]. Additionally, observed peaks and bands were caused by amino acids like phenylalanine, located at 1010 cm ^−1^, and lysine, located at 1450 cm^−1^ [18]. Conversely, typical bands for PCL were visible at 1110 cm^−1^ (vCOC, skeletal stretching) and at 1725 cm^−1^ (vC = O), which also indicated the crystalline phase [19]. The spectra for PCL:GT resembled an overlapping of collagen and PCL (see Figure 2). Despite the fact that the distinctive protein bands appeared in an attenuated form, they were still noticeable in these PCL:GT (1.2 m/s and 10.7 m/s) spectra. This applied for the strong amide I band, which was weakened, as well as the band for amide III. Furthermore, the lysine band (1450 cm^−1^) was fully overlapped by the δ(CH_2_) band of PCL (see Figure 2). A slight shift of the PCL band between 1725 cm^−1^ and 1735 cm^−1^ could be observed in both PCL:GT spectra. The comparison of the spectra for PCL:GT (1.2 m/s) and PCL:GT (10.7 m/s) revealed slight differences in the band structure around the first PCL band around 1110 cm^−1^ (see Figure 2).

### 3.2. SEM-Based Analysis 

#### 3.2.1. Fibre Scaffolds

**Figure 3:**
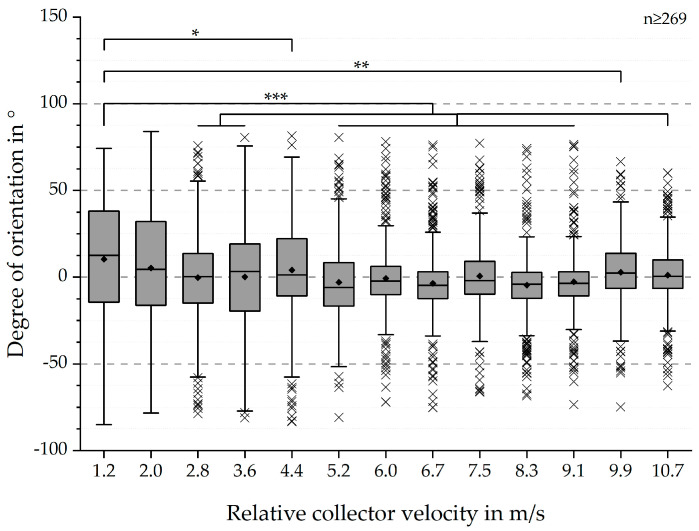
Boxplots of the degree of fibre orientation in ° for each of the 13 relative collector velocities. The resulting boxplots, including outliers, showed a trend of decreasing IQR with increasing relative collector velocity. This trend continued until 8.3 and 9.1 m/s, after which it reversed. Correspondingly, the dispersion followed a similar trend and decreased with increasing relative collector velocity. Based on the QQ-plots, indicating normal distribution, and the recorded values are independent, parametric tests were conducted. Statistical significances for all groups were investigated via one-way repeated measure ANOVAs. Mean differences between the group for 1.2 m/s and the others were analyzsed by the Dunnett post-hoc test and labelled as follows: * (*p* < 0.05), ** (*p* < 0.01) and *** (*p* < 0.001).

The analysis of fibre alignment, based on SEM-images, resulted in degrees of orientation between −85° and 81°, with calculated mean values around 0° (see Figure 3). Resulting dispersions exhibited a decrease with increasing relative collector velocity. Similarly, the IQR decreased with the same trend. The smallest IQR could be observed for 8.3 m/s and 9.1 m/s. The conducted QQ-plots suggested the application of a parametric test. Significant differences in degree of orientation were found for all relative collector velocities, except for 2.0 m/s: 4.4 m/s (* *p*-value < 0.05); 9.9 m/s (** *p*-value < 0.01); 2.8 m/s, 3.6 m/s, 5.2 m/s, 6.0 m/s, 6.7 m/s, 7.5 m/s, 8.3 m/s, 9.1 m/s and 10.7 m/s (*** *p*-value < 0.001). 

**Figure 4:**
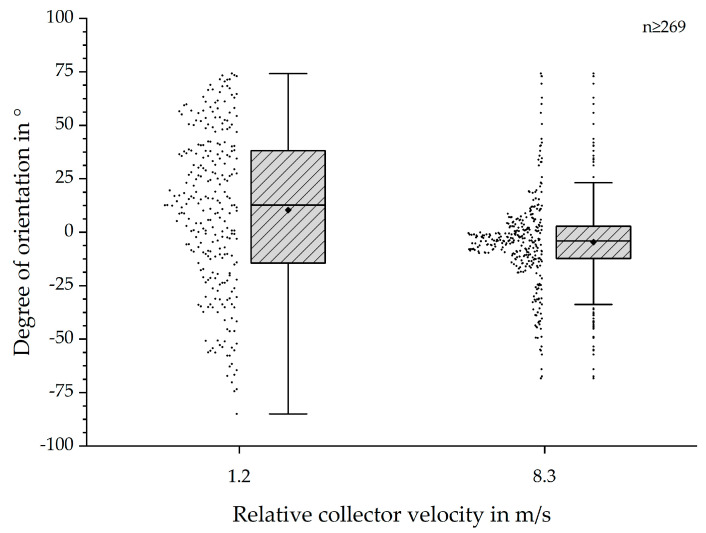
Boxplots of degree of fibre orientation in ° for 1.2 and 8.3 m/s relative collector velocity. The individually measured values are presented on the left side of each boxplot. The depicted boxplots show the results for the lowest relative collector velocity (1.2 m/s), which is defined as “control” for the Dunnett post-hoc test, and the results for 8.3 m/s without outliers. Both data sets show a distribution within a similar range, but for 8.3 m/s the majority of values is located around 0°. A much wider distribution for 1.2 m/s can be observed. The comparison of both data sets shows a decreased IQR and dispersion with increased relative collector velocity. The depicted IQR indicates normally distributed values.

In order to further evaluate the results, we compared the lowest possible and one of the highest degrees of orientation, as shown in Figure 4. The displayed values for the degree of orientation ranged from −85° to 74° and −68° to 74°. The displayed distributions showed a much higher occurrence of degrees of orientation around 0° for 8.3 m/s than for 1.2 m/s. Consequently, the dispersion and IQR of the data shows a significant decrease from 1.2 m/s to 8.3 m/s. An initial hypothesis, based on the findings of Fricke et al., stated an increased degree of orientation with increased relative collector velocity until a certain threshold. The obtained data supported this hypothesis for PCL and PCL:GT fibre scaffolds as well [9].

#### 3.2.2. Collagen Film

**Figure 5:**
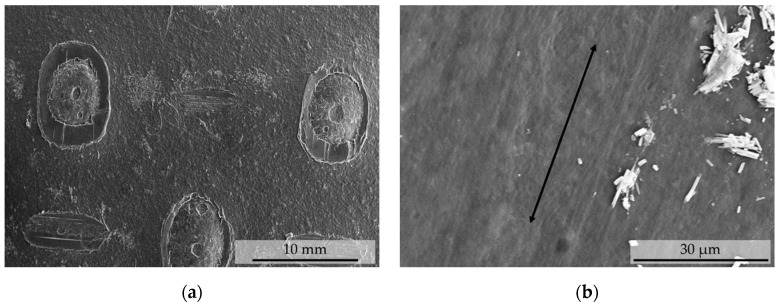
SEM images of the transparent collagen film. (**a**) shows a magnification of a factor of 35. Here the perforation of the film can be clearly seen. (**b**) shows a higher magnification of a factor of 50,000. The black arrow points in the unrolling direction of the collagen film. Probably during a rolling process, the film was stretched in this direction, which could have led to the structures visible here.

SEM images showed the expected structure of the film. In contrast to the electrospun fiber mats, the sample was not composed of individual fibers. The observed structure may be explained by the fabrication method. Presumably, the collagen was brought to the desired thickness within a extruding or milling process. During the manufacturing process, an embossing was applied. This is shown in Figure 5a. In this context, fiber orientation means the orientation of the collagen fibers themselves. In Figure 5b the same sample is seen but with a higher magnification. The black arrow shows the direction of unrolling of the transparent collagen film. The surface structure supports the hypothesis that the sample was stretched in this direction. Such stretching leads to an orientation of the collagen fibers [20,21].

### 3.3. Mueller Matrix Measurements

#### 3.3.1. Fibre Scaffolds

**Figure 6:**
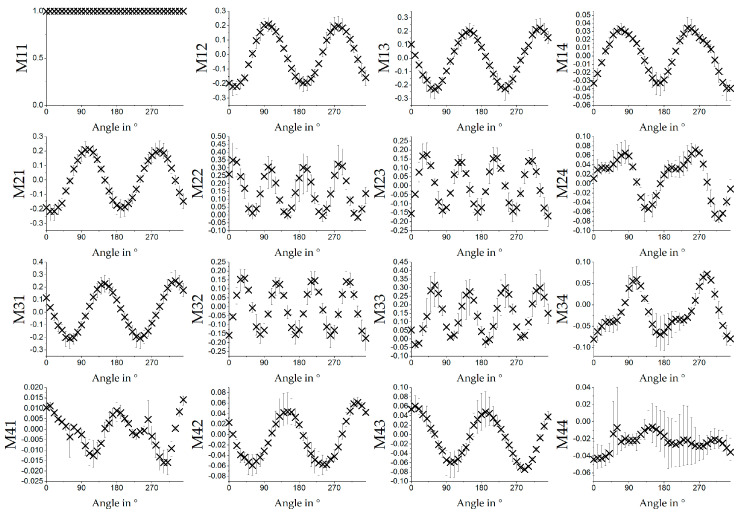
Average values of the MM images for relative collector velocity 8.3 m/s (*n* = 3; error bars: min. and max.) for different angles of the sample measured in transmission with 532 nm. The fibres are oriented horizontally at 0°.

Figure 6 shows the results of MM measurement of a sample of electrospun fibre scaffolds produced at a relative collector velocity of 8.3 m/s. Average values over the measurement of three different samples are shown. Measurement was performed using a 532 nm light source. The samples were mounted in a rotation stage and rotated in steps of 10° with respect to the orientation of the polarisation states of the MM measurement setup. At 0°, fibres were oriented horizontally. The hypothesis was that PCL and gelatin were oriented within the fibres due to the production process. While rotating the sample, angle dependent polarisation changing properties, like for example angle change of the fast axis for retardation, became visible. Changes expressed themselves in the single MM entries as a sinusoidal signal. The M11 value always equals one because all other entries were normalized to this entry. The entries M12, M13, M14, M21, M31, M42 and M43 showed a sinusoidal behavior with a period length of 180°. M22, M23, M32 and M33 showed sinusoidal behavior with a period length of 90° and M24, M34, M41 seemed to show a superposition of the two signals, which had a period length of 180° and were shifted 90° to each other. M44 showed also a somewhat periodic signal, but the result was not clearly identifiable as a sinus. In addition, the possible periodic signal was comparably smaller than the error bars. Results for M12, M13, M21, M22, M23, M31, M32 and M33 were similar to the results for pure PCL fibres (see [9]). M14 was similar for pure PCL vs PCL/GE in terms of the angle-dependent sinusoidal behavior, but the amplitude was much smaller for pure PCL (around 0.005) compared to PCL/GE (around 0.03). The other entries for the pure PCL showed no angle-dependent signal. This led us to the conclusion that the angle-dependence of the remaining entries for the PCL/GE samples was due to the gelatin content in the fiber scaffolds. For interpretation, elements M14 and M41 could be connected to the circular diattenuation, M24 and M42 to linear retardance oriented at 45° or 135° and M34 and M43 to a circular retardance [22].

**Figure 7:**
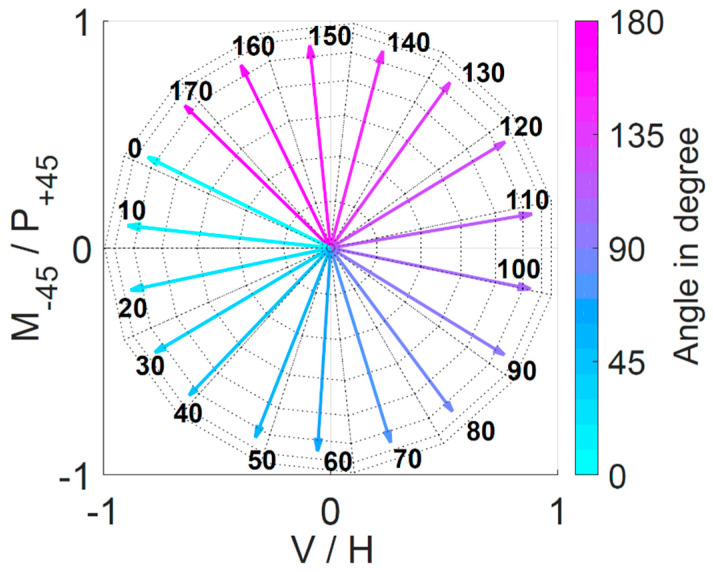
Stokes vector of the fast axis of retardation for different sample orientation. The vectors are averaged over the given sample size of three and each angle was measured twice as the sample was rotated by 370°.

As mentioned in Section 2.4, any MM can be decomposed into MMs, representing well-known optical elements to interpret the results. Figure 7 shows the results of the calculation of the Stokes vector for the fast axis of the retardance R. The vectors are shown in the equatorial plane of a Poincaré sphere. Here the vertical (V) and horizontal (H) direction faced each other as well as the −45° (M) and −45° (P). The result showed that a rotation of the sample by 10° also led to a rotation of the vector of the fast axis of the retardance by approximately 10°. This demonstrated that it was possible to obtain the orientation of the fibre scaffolds from only one MM measurement by calculation of the vector.

Compared to previous results (see [9]) this calculation seemed to be more precise for the PCL:GT fibre scaffolds compared to scaffolds made of pure PCL. For PCL, the vectors were not only in the equatorial plane resulting in a standard derivation between theoretical sample vector and vector of the fast axis of retardance of 48°. For the PCL:GT fibres, the difference in the angle between the vectors from the theoretical 10° was below 1° and was therefore considered smaller than measurement uncertainty of the manual sample positioning progress. However, the new measurement shows that the theoretically assumed vectors for the PCL were most likely not the actual fibre directions, as further discussed in Section 4. At 0°, the sample was oriented horizontally. The vector of the fast axis of retardance was nearly vertically oriented (about 13° deviation). This would support the hypothesis that the light perpendicular to the orientation of the fibres could not interact with the molecules and therefore passed through the sample more quickly.

#### 3.3.2. Collagen Film

**Figure 8:**
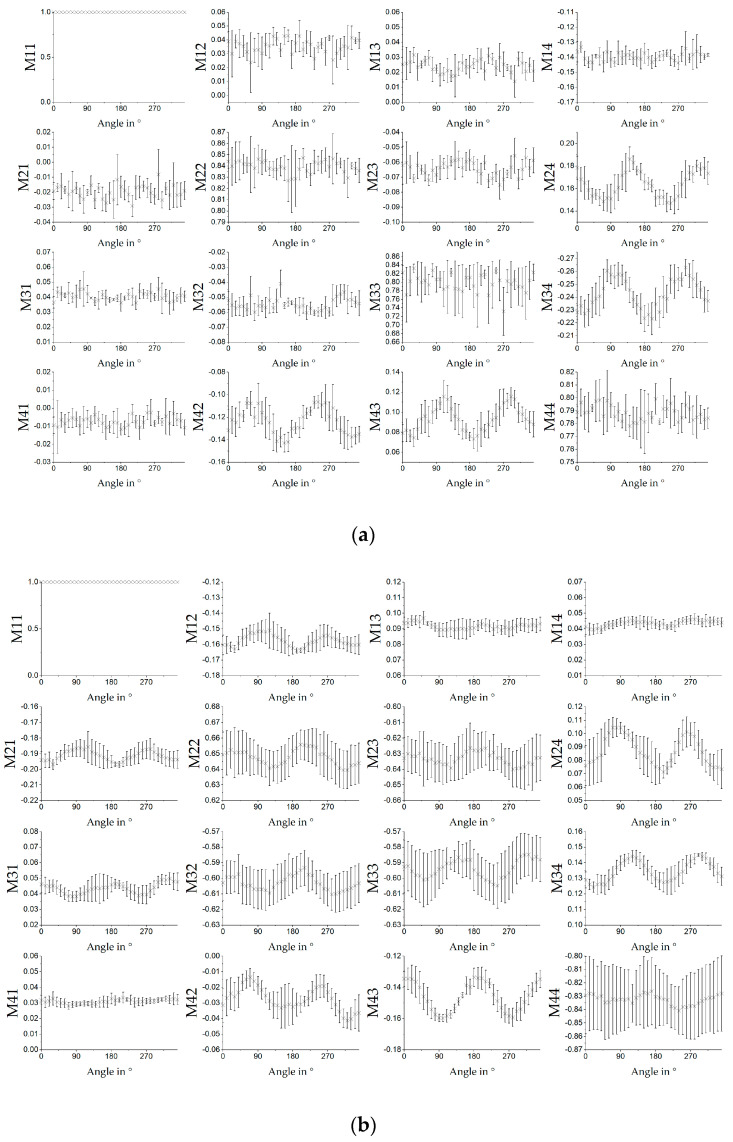
MM results for rotation of the transparent collagen film measured in (**a**) transmission and (**b**) reflection (*n* = 5; ±SD)**.** Measurement was performed with 532 nm light source.

**Figure 9:**
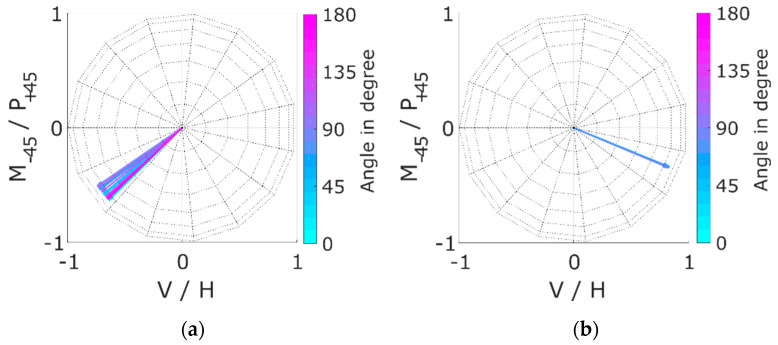
Stokes vector of the fast axis of retardation for different sample orientation of the transparent collagen film in transmission (**a**) and reflection (**b**). The vectors are averaged over a given sample size of three and each angle was measured twice because the sample was rotated by 370°.

Figure 8 shows the results of the MM measurements for different orientations of the sample. It was rotated in steps of 10°. Measurement was performed with a light source of 532 nm. Figure 8a shows the results for measurement in transmission and Figure 8b in reflection mode. The rolling direction of the sample from the roll was horizontal in relation to the measuring system at the angle of 0°. Results for transmission were made with the same experimental setup as for the results in Figure 6. Error bars indicated the standard derivation for the given sample size of *n* = 5. Compared to the results from the fibre scaffolds, the results for the collagen film showed more noise. In any case, there was a sinusoidal signal visible in M24, M34, M42 and M43 with a period length of 180°. Calculation of the Stokes vector of the fast axis of retardation are shown in Figure 9a. The Stokes vector varied between (1, −0.7, −0.7, 0) ^T^ and (1, −0.8, −0.6, 0) ^T^. It could be seen that it did not follow the sample rotation as shown for the PCL:GT fibre samples in Figure 5.

Figure 8b shows the results for the measurement in reflection mode. Here, the sinusoidal signals in M24, M34, M42 and M43 could be seen as well. In addition, sinusoidal signals could be seen in M22, M23, M32 and M33 and, with a lower amplitude, may also have been present in M12, M21 and M31. Compared with the results for electrospun fibre mats from PCL (see [9]), these results were different and much more noisy. As noticeable in Figure 9b, the Stokes vector of the fast axis of retardation is approximately (1, 0.9, −0.4, 0) ^T^ for all measurements. Compared with the orientation of the vector of the fast axis for the retardation for PCL:GT fibres shown in Figure 7, the orientation did not correlate with the sample orientation. However, if sample properties are known, orientation could still be obtained from the data by performing a reference measurement. For example, in M24 and M43 from Figure 8a,b the combination of the values would allow conclusions to be drawn about the orientation of the sample, up to a certain precision, as also supported by the standard deviation.

## 4. Discussion

We were able to point out, that a non-contact and thus non-destructive measurement of the orientation of collagen- and gelatin-containing samples is generally possible. The obtained spectral data showed distinct differences in molecular structure for the samples used in this study: PCL, PCL:GT (1.2 m/s and 10.7 m/s) and collagen film. The recorded spectrum for the collagen film indicated a strong presence of proteins, unchanged in their conformation. The appearance of the most prominent peak for collagen, located at 1667 cm^−1^, indicates that the film indeed consisted of collagen and not of denaturized proteins. The comparison with the PCL:GT fibre scaffolds shows some similarities, probably caused by the fact that gelatin is derived from native collagen [23]. Correspondingly, these similarities reveal that the gelatin is not altered by the electrospinning process. The still observable bands for amide II and III (1220 cm^−1^ and 1280 cm^−1^) distinguish the PCL:GT blends from the pure PCL. Furthermore, the characteristic band for PCL at 1110 cm^−1^ changes with increasing the relative collector velocity from 1.2. m/s to 10.7 m/s and thus, indicating changes in the PCL:GT fibre scaffolds properties in comparison to pure PCL. Further analysis, both qualitative and especially quantitative, are necessary to determine the actual influence of the relative collector velocity on these bands and therefore fibre scaffold properties. However, a slight shift of the PCL band between 1725 cm^−1^ and 1735 cm^−1^ was observed in both PCL:GT spectra. Consequently, an effect on the ratio of amorphous to crystalline phases of the PCL in PCL:GT can be assumed [19]. The qualitative Raman analysis illustrates an impact of gelatin on the PCL structure and the other way around, within the blended fibre scaffold. 

**Figure 10:**
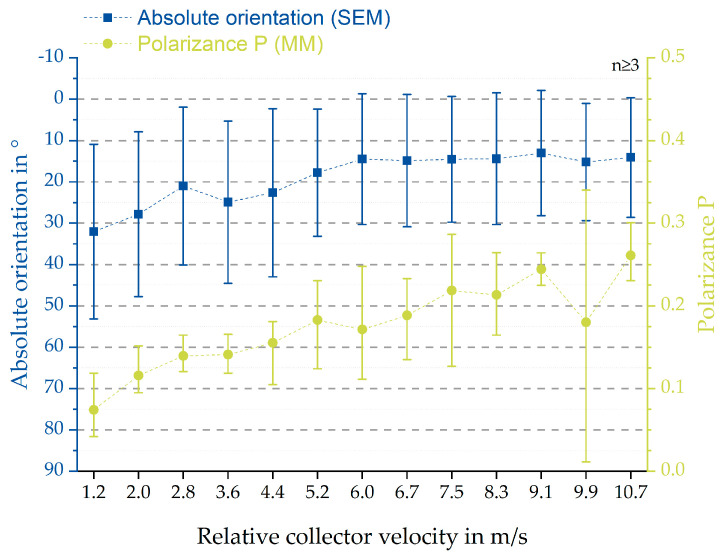
Correlation between polarizance P (resulting from MM measurements) and absolute orientation (measured SEM-based) of the PCL/GT fibre scaffolds. The green circles represent the mean values for the polarizance P with error bars (min. and max.) whereas the blue squares show the results for the absolute orientation with error bars (±SD).

The SEM-based analysis still bears several factors increasing errors with regards to the mean values of the fibre alignment (see Figure 10) [9]. However, the statistical analysis and presented boxplots (see Figure 3 and Figure 4) clearly indicate a significant increase of the degree of fibre orientation with increasing relative collector velocity. Correspondingly, the initially hypothesised threshold was reached around 8.3 m/s, represented by the smallest IQR for 8.3 m/s and 9.1 m/s. In spite of the smallest IQR for these relative collector velocities, the number of outliers is higher than for all other groups. However, the reason for this observation does probably not originate from the measurement method but from the statistical analysis. The definition of the whiskers is 1.5 times the IQR. Hence, closer distributions will lead to smaller dispersion and therefore apparently more outliers. So, there is no simple answer to the question whether these results represent statistical anomalies or the influence of the measurement protocol [9]. Overall, these results are consistent to those obtained by Fricke et al. indicating an influence of the relative collector velocity, not being limited only to PCL fibre scaffolds. Further investigations have to be conducted to generate a calibration curve for generic materials. 

Furthermore, the analysed fibres exhibited diameters up to 450% smaller than for pure PCL (see Supplementary Materials). Nevertheless, the observed trend (see Figure 10) with regard to degree of orientation in dependence of relative collector velocity, is similar to the one described by Fricke et al. [9].

To demonstrate the potential of MM measurements for samples that are not electrospun, the results were compared with the measurements of a transparent collagen film. The correlation between the absolute orientation and polarizance P is clearly depicted in Figure 10. The MM measurement of PCL:GT showed the dependence of MM entries on the orientation of the sample. Compared with previous results for PCL [9], a significant difference can be seen. For PCL:GT, in addition, MM entries are sensitive to rotation of the sample, which are probably related to circular diattenuation. This may be explained by the chirality of the gelatin (applies also for collagen) [24,25,26,27] component while pure PCL has no chiral structure. While results for PCL:GT also show an angle dependence of the entries connected with linear retardation and diattenuation [22], results for these entries are not as clear for the transparent collagen film. Here mainly MM entries that are connected to circular retardance [22], were sensitive to rotation of the sample. For the PCL:GT entries like M24, M34 and M41 (see Figure 6), the data seem to show a superposition of two signals. A reason for this could be the different optical properties of gelatin and PCL. Another explanation could be a bimodal distribution of the orientation of the fibres, which is less likely because no supporting observations were made during the measurements (see Figure 4). Further calculation of the fast axis of the retardance show that the angle of the sample is strongly connected to the angle of the fast axis for the PCL:GT samples. Here, one MM measurement is sufficient to measure sample orientation. Measurement is more precise compared to previously shown results for PCL fibres [9]. The angle of the fast axis is not directly connected to the orientation of the fibres as assumed. In particular, it was assumed that fibres are oriented in the direction of rotation of the collector (see Figure 1). Because light can interact with electrons, which are capable of moving in direction of orientation of the molecules, the fast axis of retardation should be perpendicular to the fibre orientation. As fibres were oriented horizontally at 0°, the Stokes vector of the fast axis of retardation should be vertical. However, there is a difference of about 13°. This difference indicates that the fibres are not oriented in the direction of the rotation of the collector but are oriented with a certain angle due to a side movement during the spinning process. The side movement could be a result of an inhomogeneous electric field of the apparatus. However, the calculation is not suitable to calculate sample orientation of the transparent gelatin film. Here, the orientation of the sample can only be extracted if the amplitude of the signal for the angle dependent MM entries is known.

## 5. Conclusions

The performed MM measurements are a promising method to measure orientation of bio-polymer structures in skin phantoms or the skin itself. Results showed that angle dependence of the MM entries depend strongly on the samples investigated. That demonstrates the strength of the MM approach, which is suitable to measure any polarisation altering property of the sample. The orientation of PCL:GT samples can be measured with only one MM measurement by calculating the Stokes vector of the fast axis of retardation without prior knowledge of the amplitude of the signal. For the transparent collagen film, orientation can be obtained by using prior knowledge of the amplitude of the angle dependent signal in the MM entries M34 and M43. In order to apply this measurement method to different materials, the algorithms developed might have to be adjusted. Ongoing studies are investigating the power of other decomposing methods and further characterisation of collagen samples within one measurement. 

The presented results of Raman spectroscopy, SEM-based analysis of the degree of fibre orientation and MM measurements strongly indicate the suitability of PCL:GT fibre scaffolds and collagen containing samples for calibration of skin disease detection and evaluation by measuring the orientation of given structures and the presence of chiral molecules, which manifest themselves by an angle dependent signal in certain MM entries.

## 6. Patents

European patent “Method for the morphological characterization of fiber mats by polarimetry” pending (No. EP19197842.8).

## Supplementary Materials

The following are available online at https://www.mdpi.com/2073-4360/12/6/1400/s1, Figure S1: Boxplots of fiber diameters in µm for each of the 13 relative collector velocity. The resulting boxplots, including outliers, show no specific trend for the IQR with increasing relative collector velocity. Likewise, the dispersion does not express a specific trend either. Based on the results of the QQ-Plots, and the recorded values been independent, parametric tests were conducted. Statistical significances for all groups were investigated via one-way repeated measures ANOVA, indicating significant differences. Mean differences between the group for 1.2 m/s and the others were analyzed by Dunnett post-hoc test and labeled as follows: * (*p* < 0.05), ** (*p* < 0.01) and *** (*p* < 0.001).
