# Supplementary material for: Mueller Matrix Analysis of Collagen and Gelatin Containing Samples Towards More Objective Skin Tissue Diagnostics
Source: Polymers (Basel). 2020 Jun 22;12(6):1400. doi: 10.3390/polym12061400 (PMC7361993; doi:10.3390/polym12061400)
Supplement: Supplementary file 1 [file polymers-12-01400-s001.pdf]

Supplementary Material

For the Article: Mueller matrix analysis of collagen and gelatin containing samples towards enabling more objective skin disease diagnostics

Content

This Supplementary Material contains additional measurements performed for the fiber scaffolds presented in the main part. Fibers diameters were investigated to fully characterize the fabricated scaffolds.

1. Analysis of fiber diameters

We used the method previously published by Fricke 2019 et al. [1]. In this study, a total of 9 SEM images (three of each fiber scaffold) per relative collector velocity were taken and analyzed. The resulting fiber diameters are displayed as boxplots with outliers (see Figure 1).

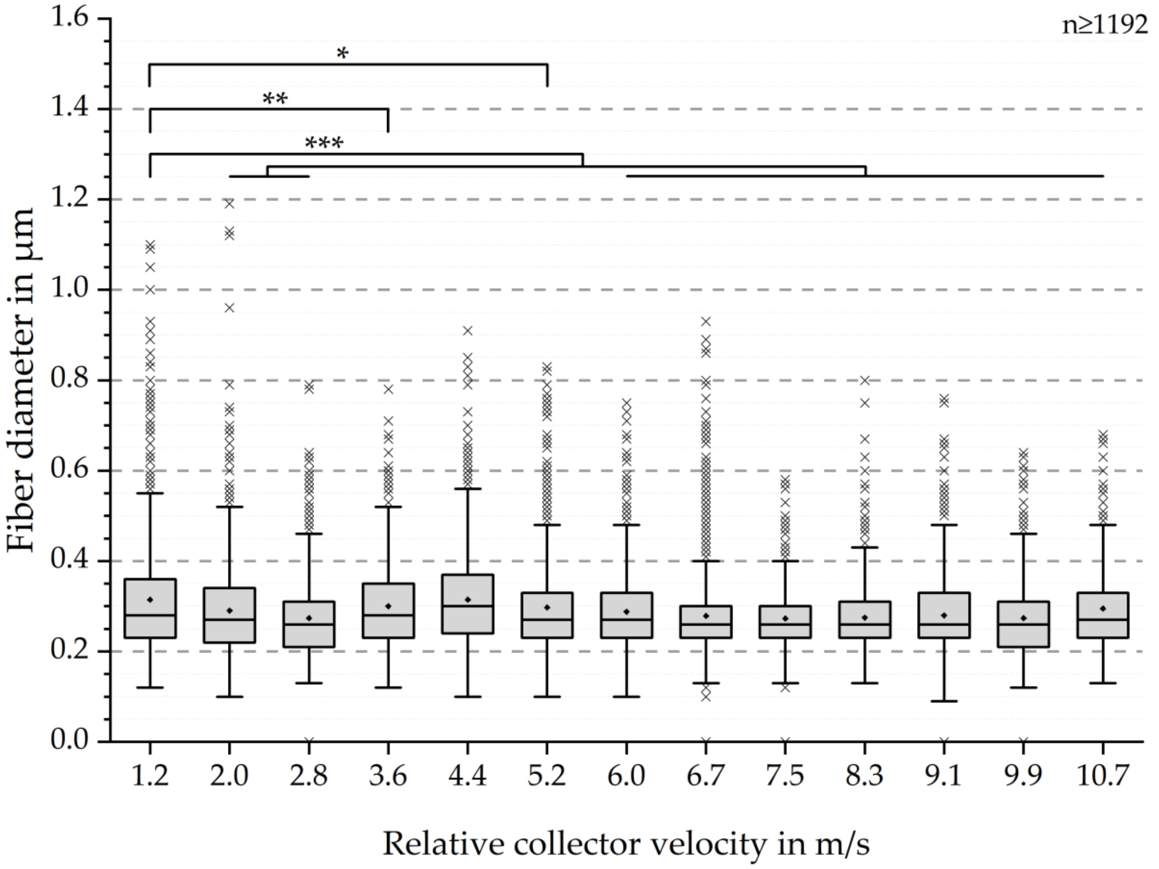

Figure 1 Boxplots of fiber diameters in µm for each of the 13 relative collector velocity. The resulting boxplots, including outliers, show no specific trend for the IQR with increasing relative collector velocity. Likewise, the dispersion does not express a specific trend either. Based on the results of the QQ-Plots, and the recorded values been independent, parametric tests were conducted. Statistical significances for all groups were investigated via one-way repeated measures ANOVA, indicating significant differences. Mean differences between the group for 1.2 m/s and the others were analyzed by Dunnett post-hoc test and labeled as follows: * ($p < 0.05$), ** ($p < 0.01$) and *** ($p < 0.001$).

The Interquartile Range (IQR) is defined as 50% of the measured data and indicated via a closed box. While the displayed whiskers represent 1.5 times the IQR, the line dividing the IQR, represents the median value. All values located outside of 1.5 x IQR are displayed as individual crosses. These values

are defined as “outliers” with regards to the IQR, but are not tested to be extreme values. The mean value is displayed as rhombus. Conducted QQ-Plots suggested the usability of parametric tests. In order to determine differences between the groups, one-way repeated measures ANOVA was conducted. Subsequently, Dunnett post-hoc tests were performed to identify differences between individual groups. Differences were considered significant at $p < 0.05$ (*), $p < 0.01$ (**) and $p < 0.001$ (***). All data was analyzed using a statistical analysis software (Origin 2018b, OriginLab Corporation, Northampton, Massachusetts, USA).

As shown in Figure 1, the manufactured fiber scaffolds consist of PCL:Gelatine fibers with diameters ranging from 0.1 µm to 1.19 µm. Correspondingly, mean values from 0.27 µm up to 0.31 µm were calculated. The displayed dispersion, range between the whiskers, vary between the groups for the thirteen different relative collector velocities. Additionally, outliers can be observed for all groups. Despite the absents of an observable trend, the conducted Dunnett post-hoc test resulted in significant differences between the lowest relative collector velocity (1.2 m/s) and all the other groups except for 4.4 m/s. The analyzed samples for 2.0 m/s, 2.8 m/s, 6.0 m/s, 6.7 m/s, 7.5 m/s, 8.3 m/s, 9.1 m/s, 9.9 m/s and 10.7 m/s showed the highest significant differences with a p-value < 0.001 (***). The results for 3.6 m/s exhibited differences with a p-value < 0.01 (**) and for 5.2 m/s the analysis showed the lowest significance with a p-value < 0.05 (*). The statistical analysis showed an influence of the relative collector velocity on the fiber diameter for eleven out of twelve studied groups (1.2 m/s set as the reference).

Further investigations need determine whether the differences are originated in the adjustment of the relative collector velocity and therefore the electrospinning process or the measurement protocol. Compared to PCL-based fiber scaffolds, the measured diameters are up to 450% smaller [1]. Due to the SEM-based method, this fact could impact the results significantly. As suggested by Fricke 2019 et al, an automated fiber diameter measurement like developed and presented by Hotaling 2015 et. al., could be a solution to this problem [2].

2. References

1. D. Fricke, A. Becker, L. Jütte, M. Bode, D. de Cassan, M. Wollweber, B. Glasmacher, and B. Roth, “Mueller Matrix Measurement of Electrospun Fiber Scaffolds for Tissue Engineering,” Polymers **11** (2019).
2. N. A. Hotaling, K. Bharti, H. Kriel, and C. G. Simon, “DiameterJ: A validated open source nanofiber diameter measurement tool,” Biomaterials **61**, 327–338 (2015).
